# Human stem cell neuronal differentiation on silk-carbon nanotube composite

**DOI:** 10.1186/1556-276X-7-126

**Published:** 2012-02-14

**Authors:** Chi-Shuo Chen, Sushant Soni, Catherine Le, Matthew Biasca, Erik Farr, Eric Y-T Chen, Wei-Chun Chin

**Affiliations:** 1 Bioengineering Program, School of Engineering, University of California, Merced, CA, USA; 2 School of Nature Sciences, University of California, Merced, CA, USA

**Keywords:** CNT, silk, fibroin, human stem cell, neuron differentiation, scaffold

## Abstract

Human embryonic stem cells [hESCs] are able to differentiate into specific lineages corresponding to regulated spatial and temporal signals. This unique attribute holds great promise for regenerative medicine and cell-based therapy for many human diseases such as spinal cord injury [SCI] and multiple sclerosis [MS]. Carbon nanotubes [CNTs] have been successfully used to promote neuronal differentiation, and silk has been widely applied in tissue engineering. This study aims to build silk-CNT composite scaffolds for improved neuron differentiation efficiency from hESCs.

Two neuronal markers (β-III tubulin and nestin) were utilized to determine the hESC neuronal lineage differentiation. In addition, axonal lengths were measured to evaluate the progress of neuronal development. The results demonstrated that cells on silk-CNT scaffolds have a higher β-III tubulin and nestin expression, suggesting augmented neuronal differentiation. In addition, longer axons with higher density were found to associate with silk-CNT scaffolds.

Our silk-CNT-based composite scaffolds can promote neuronal differentiation of hESCs. The silk-CNT composite scaffolds developed here can serve as efficient supporting matrices for stem cell-derived neuronal transplants, offering a promising opportunity for nerve repair treatments for SCI and MS patients.

## Background

There are about 250,000 to 400,000 patients in the US suffering from spinal cord injury [SCI] [1], usually due to trauma or traffic accidents, which could lead to death or life-long paralysis. According to the National Multiple Sclerosis Society, there are about 400,000 multiple sclerosis [MS] patients in the US, and this number is steadily growing: about 200 people are diagnosed each week. Currently, there is no effective cure for SCI or MS since adult humans do not fully regenerate their damaged neurons and axons. The inability for the body to regenerate and re-innervate target neuronal axons greatly limits therapy feasibility [1,2]. The unique abilities of human embryonic stem cells [hESCs] - namely, their self-renewal and potency - hold great promise for regenerative medicine. For SCI and MS patients, the capacity of hESCs to differentiate into specific neuronal lineages through effective induction is highly encouraging. The unique appeal of hESC-based transplantation for SCI and MS is the possibility of those transplanted cells to repair damaged neuronal tissues. However, the harsh microenvironment and the lack of supportive substrates during transplantation result in a low survival rate of transplanted cells and diminish the feasibility of stem cell-related cell therapy [3]. In regenerative medicine and tissue engineering, both naturally derived and synthetic materials have been extensively explored and provided their respective advantages [4]. For instance, nanofibers incorporated with the pentapeptide epitope, isolucine-lysin-valine-alanine-valine, were constructed to induce rapid differentiation of cells into neurons. Biomaterials synthesized with synthetic or natural polymers were fabricated to facilitate the complex tissue formations [4-9]. Generally, due to their inherent properties of biological recognition, extracted natural proteins present better cell-triggered proteolysis degradation and biocompatibility; synthetic materials provide more flexible material properties with specific designs. In this study, we aimed to integrate natural silk fibroin protein with synthetic carbon nanotubes [CNTs] to construct scaffolds for neuronal developments.

CNTs are a conductive biomaterial with sizes comparable to extracellular matrix molecules such as collagens and laminins, which have been reported to favor neuronal growth [10,11]. In addition, due to their excellent mechanical strength and flexibility, CNTs can contribute to the structural integrity of scaffolds. Substrates prepared with CNTs have been demonstrated to be biocompatible and can support neuronal growth and differentiation [10]. It has also been proposed that neurons grown on a CNT meshwork displayed better signal transmission, possibly due to tight contacts between the CNTs and neural membranes, favoring electrical shortcuts [12]. All of the above characteristics make CNTs a promising biomaterial to repair damaged neuronal tissues.

Silks are natural polymers (protein) that have been widely used as biomaterials for many years. Fibroin protein is extracted from silk (*Bombyx mori*), consisting of 90% of amino acids such as glycine, alanine, and serine. Various ratios of amino acids are distributed on the supramolecular structure of fibroin, consisting of a hydrophobic heavy chain (350 kD to 370 kD) and hydrophilic light chain (25 kD) [13,14]. Due to its mechanically robust and flexible nature in thin film form, biocompatibility, and *in vivo *reabsorbing and water-dissolvable properties, fibroin protein has been used as a building material for scaffolds for various tissue engineering applications and stem cell researches [8,15-18]. For instance, fibroin scaffolds have been successfully applied to human mesenchymal stem cell differentiation, especially for ligament, bone, or cartilage tissue engineering [17,19]. In addition, successful bio-integrated electronics has been developed based on dissolvable silk fibroin films [20].

Unmodified CNTs tend to aggregate rather than disperse in aqueous solutions due to their hydrophobic nature. These heterogeneous aggregations of CNTs not only bring about difficulties in scaffold preparation, but also limit their applications. In an effort to resolve this problem, various surfactants were adapted to disaggregate and uniformly disperse CNTs in different solvents. However, the bio-toxicity of residuals still remains as one of the major concerns for cell scaffolding fabrication [21-23]. Since fibroin consists of 75% of nonpolar hydrophobic amino acids [14], it has been shown that the amphiphilic fibroin protein can effectively serve as a dispersant for CNTs [24]. Here, we used fibroin extraction to disperse CNTs homogeneously to build silk-CNT composite scaffolds. This study aims to combine the unique advantages of these two biocompatible materials to build silk-CNT scaffolds in order to acquire sufficient neuronal differentiation efficiency from hESCs for effective neuronal cell transplantation.

## Methods

### Silk fibroin preparation

Based on the protocol published by Kaplan et al. [7,8], *B. mori *silk was in boiling 0.02 M Na_2_CO_3 _(Sigma-Aldrich, St. Louis, MO, USA) for 1 h and rinsed thoroughly with deionized [DI] water to remove sericin protein associated with fibroin. The washed silk was then dissolved in 9.3 M LiBr (Fisher Scientific, Pittsburgh, PA, USA) for 3 h at 60°C. The fibroin solution was then dialyzed (MWCO 1,000, Spectrum Laboratories, Inc., Rancho Dominguez, CA, USA) in DI water for 48 h. Following which, the silk solution was centrifuged at 800 × *g*, and the supernatant was collected [25].

### Silk-CNT scaffolds and poly-l-ornithine coating

Multi-wall CNTs [MWCNTs] (Nano-Lab, Waltham, MA, USA) were dispersed in DI water and sonicated for 2 h to help disperse the MWCNT. Glass micro-coverslips were boiled in a mild surfactant for 30 min and rinsed with DI water. The coverslips were then washed with 2 N HCl overnight and cleaned with DI water. The concentration of the MWCNT/silk mixture was 1 mg/ml MWCNT in 2 wt.% silk fibroin solution. For the preparation of silk scaffolding, 600 μl of 2 wt.% silk fibroin solution was deposited on the coverslip surface at 60°C. Silk-CNT scaffolds were prepared with the silk-CNT mixed solution following a similar protocol. In order to increase cell attachment on silk fibroin [26], laminin (20 μg/ml, Sigma-Aldrich, St. Louis, MO, USA) was used to coat the scaffold surfaces [27]. Poly-L-ornithine [PLO] (Sigma-Aldrich, St Louis, MO, USA), a common substrate for neuronal differentiation, was used as the control substrate coating. PLO solution (0.1 mg/ml) was applied to the coverslip surface and incubated at 37°C overnight. Excess PLO solution was aspirated; then, the surface was rinsed with DPBS before use [27]. Silk-CNT substrates were exposed to UV for 1 h for sterilization purposes.

### Maintenance and differentiation of human embryonic stem cells

H9 hESC lines from Wicell (Madison, WI, USA; passage 32 to 55) were cultured on feeder layers of mitomycin C-treated mouse embryonic fibroblasts [MEFs] as described in our previous study [13]. The medium was changed daily, and differentiated cells were moved manually after 7 days.

The hESC cell colonies were detached from the MEF feeder layer with dispase (1 U/ml) and transferred to ultra-low contact wells. The suspended hESCs aggregated as an embryoid body [EB] and was allowed to grow for 4 to 6 days before plating on substrates. With respect to the influence of cell density on differentiations, seven to ten EB cell aggregations were seeded onto each PLO, silk, and silk/CNT substrates, with a neuron induction medium consisting of F12/DMEM, N2 supplement, and FGF2 (20 ng/ml). The medium was changed once daily for the first 2 days and then once every other day.

### Immunocytochemistry and fluorescence measurements

We stained cells using β-III tubulin (Millipore Co., Billerica, MA, USA) as a marker for neuronal differentiation with a ratio of 1:500, nestin (Millipore Co., Billerica, MA, USA) as markers for motor neuron progenitor [10], and DAPI (Invitrogen, Carlsbad, CA, USA) as nuclei markers. Cells were fixed with 4% paraformaldehyde on the seventh day for immunostaining.

Images were taken with a Nikon Eclipse TE2000-U fluorescent microscope (Nikon, Tokyo, Japan). Fluorescence intensities and axon lengths were quantified using an image analysis software (SimplePCI, Compix Inc., Sewickley, PA, USA). Statistical analysis was performed using the paired Student's *t *test.

### Scanning electron microscopy

Scanning electron microscopy [SEM] was used to investigate the substrate degradation and morphology of cells grown on the different substrates. The cells were fixed with 4% paraformaldehyde in PBS at 4°C for 20 min, followed by a series of ethanol dehydration. Carbon dioxide critical point drying was preformed to avoid specimen distortion during the drying process. The specimens were sputter-coated with a 500-Å gold thin film and examined using FEI Quanta 200 ESEM (FEI Co., Hillsboro, OR, USA).

## Results and discussion

### Flexible silk-CNT scaffold

**Figure 1:**
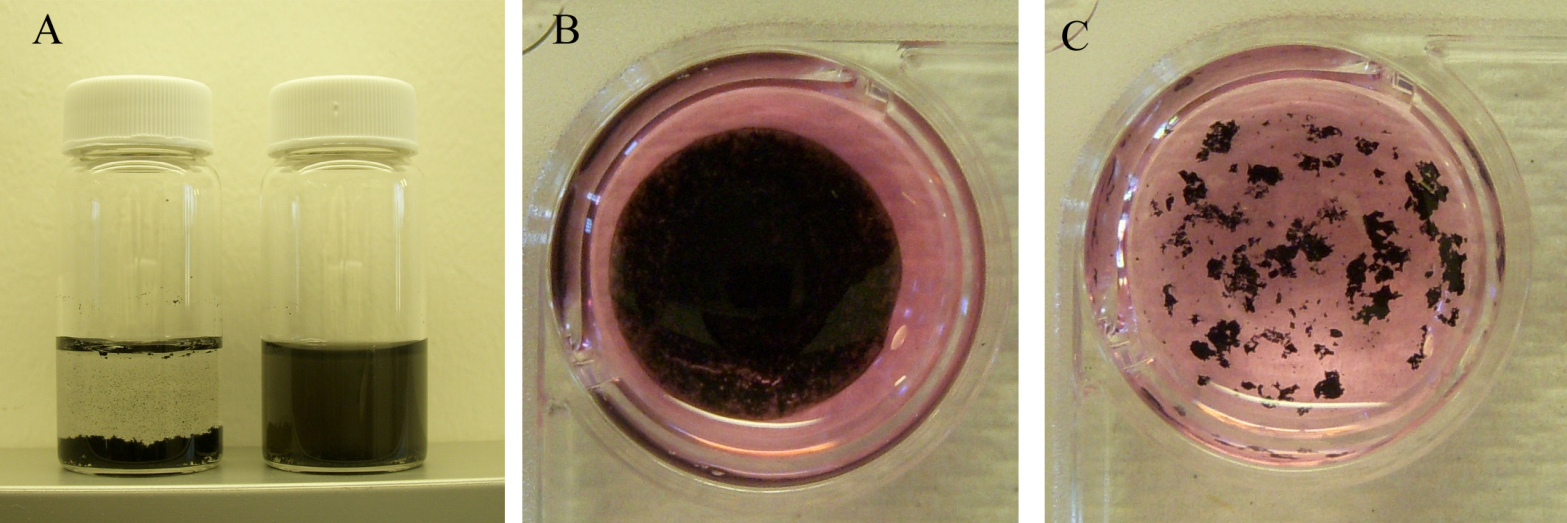
**Silk dispersion and silk-CNT scaffolding**. (**a**) The MWCNT dispersions in DI water (left) and silk fibroin solution (right). MWCNTs form a homogeneous and stable solution without sedimentation in silk fibroin solutions (right); on the contrary, they aggregate into large clusters in water (left). (**b**) Silk fibroin provides stable matrices to hold the CNTs in aqueous environments. (**c**) MWCNTs form large aggregates in aqueous solution (stem cell culture media).

The MWCNT solution exhibits a homogeneous distribution in 2 wt.% of silk fibroin solution (Figure 1a). Silk-CNT scaffolding was prepared as described, and the silk fibroin film can be peeled from the glass substrate after desiccation. Compared to the CNT embedded in fibroin matrix, CNTs deposited along the glass surface resulted in aggregated clusters in the cell culture medium (Figure 1b,c).

CNTs have been demonstrated to stimulate neuronal differentiation [6,8]; however, CNTs are easily disintegrated without supporting matrices and require delicate handling and intensive labor. Here, we used fibroin to provide mechanical and structural support for CNT-based scaffolds (Figure 1b). The amphiphilic properties of natural silk fibroin protein can not only disperse CNTs, but also form a polymer matrix to hold CNTs within its polymer network. In comparison to other hydrogels used in tissue engineering, the fibroin matrix can provide sufficient mechanical strength for transplant applications [28].

### hESCs grown on silk-CNT scaffold

**Figure 2:**
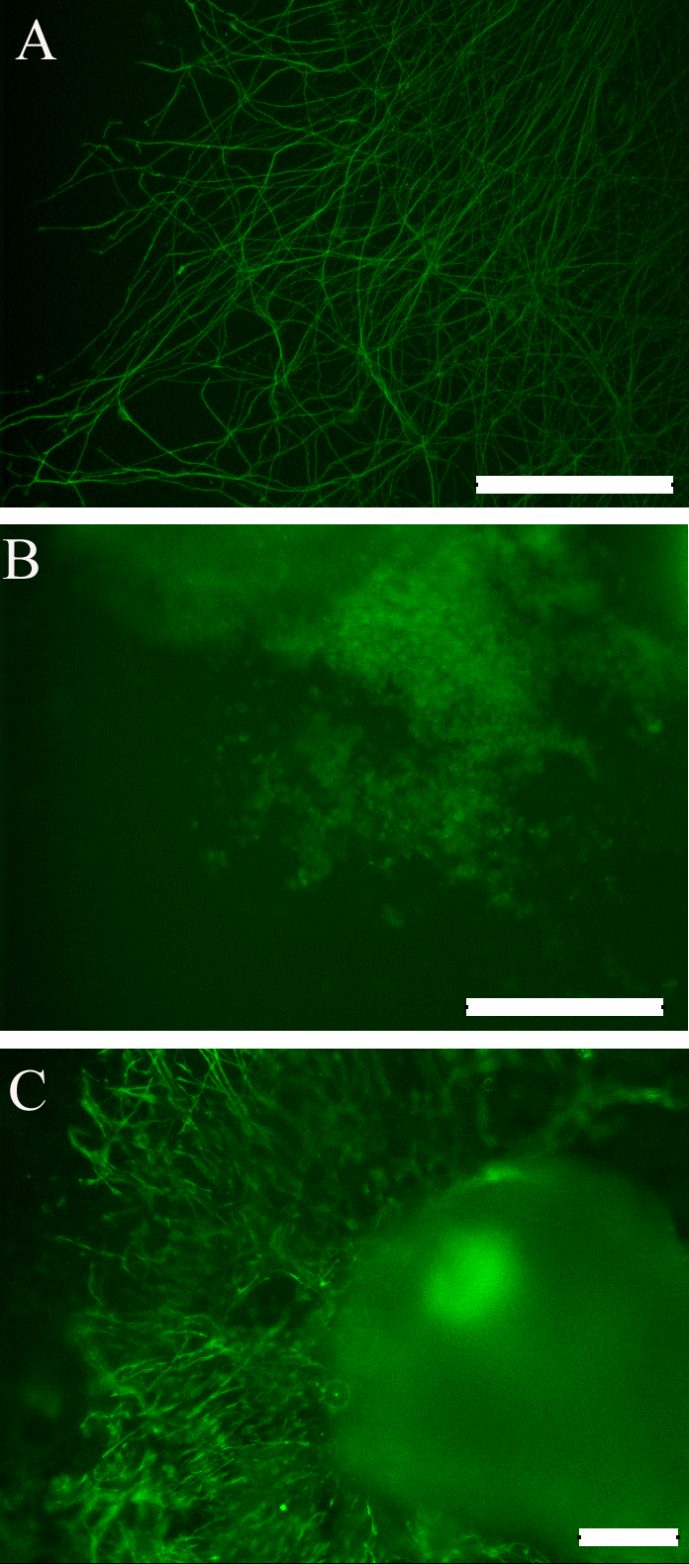
**Neuronal marker expression**. Neuronal marker, β-tubulin III, expression of hESC cultured on (**a**) PLO exhibiting long two-dimensional axonal development with lower density, (**b**) silk scaffolds exhibiting some cell migration along with negligible axonal projections, and (**c**) silk-CNT scaffolds demonstrating three-dimensional axonal elongation as well as cell migration. Scale bar, 200 μm.

In our study, a neuronal marker, β-III tubulin, was used to label differentiated neural cells from hESCs cultured on silk-CNT scaffolds, silk scaffolds, and PLO substrates [29]. Distinct neuron somas and axon shootings on silk-CNT substrates were observed (Figure 2c). Cells on silk scaffolds exhibited limited neuronal differentiation. The standard substrate for neuronal differentiation, PLO, supported moderate neuronal differentiation compared to silk-CNT scaffolds. Longer axons with higher density were found on silk-CNT composite scaffolds compared with those on the silk substrate or PLO coating (Figure 2a).

### Neuronal differentiation efficiency with image analysis

**Figure 3:**
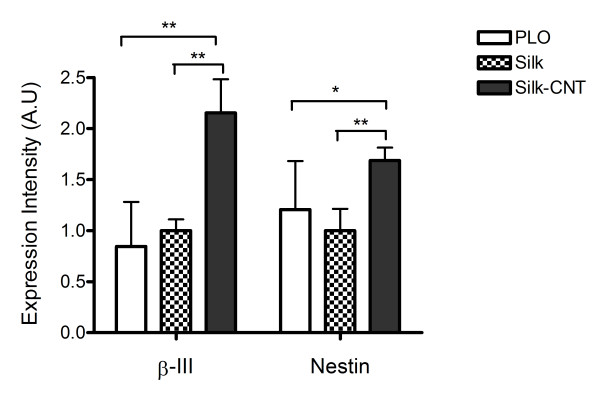
**Expression level of β-III tubulin and nestin on PLO, silk, and silk-CNT composite substrates**. Expression intensity of β-III tubulin and nestin observed with fluorescence microscopy. Silk-CNT scaffolds exhibited maximum β-III tubulin expression, while nestin expression exhibited a similar trend. Single asterisk represents *P *< 0.01, and double asterisks represent *P *< 0.001.

**Figure 4:**
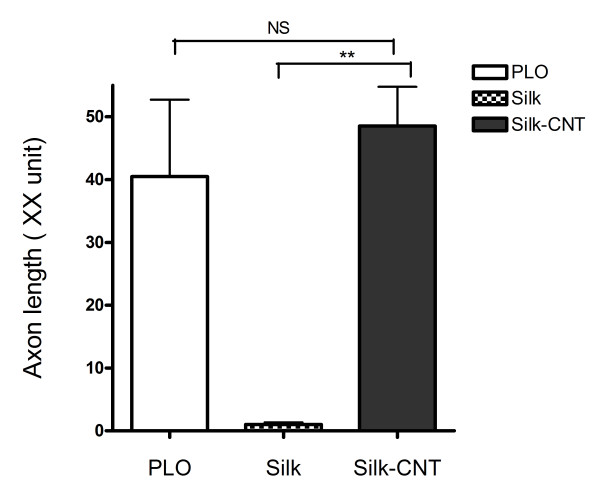
**Axonal length on PLO, silk, and silk-CNT composite substrates**. Axonal length measurements with β-III tubulin fluorescence images. PLO and silk-CNT substrates demonstrated similar axonal length; however, silk scaffolds induce very limited axonal length growth. Double asterisks, *P *< 0.001; NS, no statistical significance.

Two neuronal markers (β-III tubulin and nestin) were used to further determine the hESC differentiation efficiency. Here, β-III tubulin was used to represent the mature differentiated neurons, and nestin represented the neuron precursors [29,30]. The image analysis results showed that the expression level of β-III tubulin and nestin was highly upregulated in hESCs grown on the silk-CNT substrate (*P *< 0.001, compared to the expression level of cells grown on the PLO substrate, Figure 3). However, fewer neuron cell bodies and little to no axon shootings were found on substrates derived from silk alone compared to cells on the silk-CNT substrate. The axonal lengths of neurons grown on different substrates were measured using the image analysis software as described. Results demonstrated that there is no significant difference between the axon length of cells grown on silk-CNT and PLO substrates (*P *= 0.08), but significantly shorter axons were associated with cells on the silk substrate (Figure 4).

### Cell morphology and silk-CNT substrate degradation

**Figure 5:**
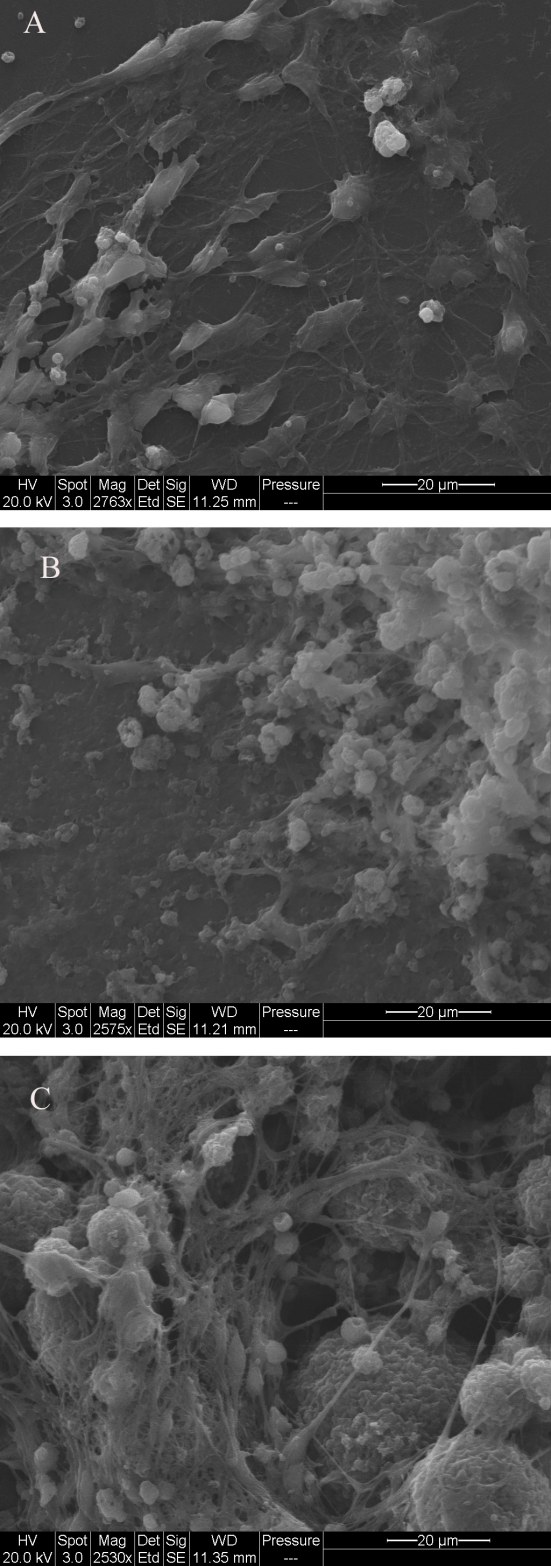
**SEM images of hESCs on various substrates**. SEM images of (**a**) cells cultured on PLO exhibiting a flat morphology and two-dimensional axonal connections, (**b**) cells cultured on silk scaffolds demonstrating three-dimensional structures and cell migration, and (**c**) cells cultured on silk-CNT scaffolds demonstrating three-dimenstional axonal connections and silk-CNT matrix degradation.

We used SEM to investigate the cell morphology on different culturing substrates. Stem cells were grown on various substrates for 7 days with the neuron-inducing medium before fixation. Obvious axon extensions were observed with attached differentiated neuronal cells (Figure 5a,b,c). In comparison to the cells cultured on silk-CNT scaffolds, most of the cells exhibited a flatter morphology with axonal connections limited to two dimensions. Although cells cultured on the PLO-coated glass exhibited long axonal extensions, the spatial density distribution of axonal networks was lower than that on silk-CNT. Cells grown on the silk substrate exhibited three-dimensional [3-D] morphology; however, the cells demonstrated limited axonal extensions along with unstable adhesion to the scaffold. In the case of the cells cultured on silk-CNT scaffolds, dense complex three-dimensional axonal bundle networks were observed. The SEM images further confirmed the results of the image analysis.

**Figure 6:**
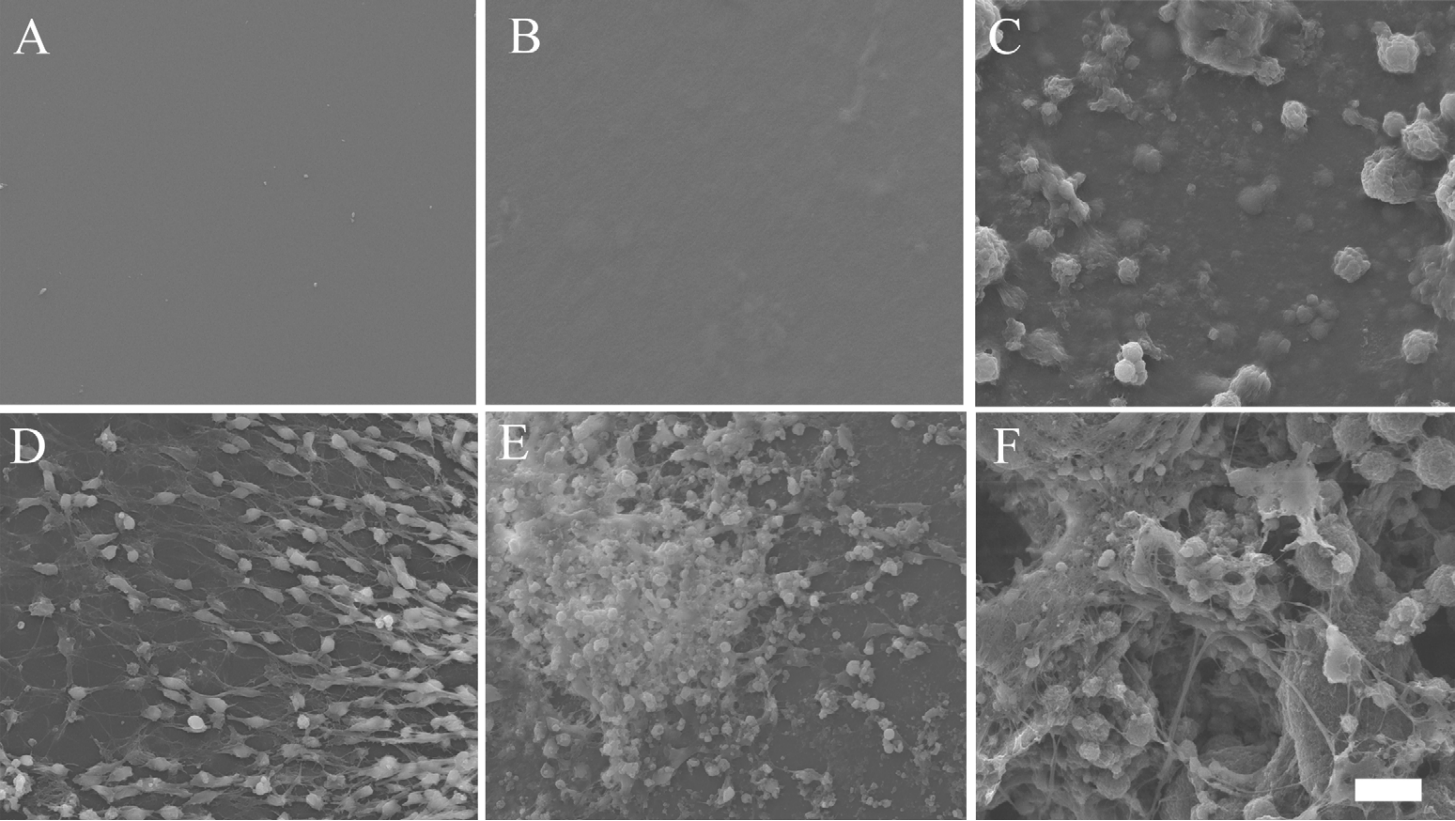
**SEM image of silk-CNT composite**. SEM images of PLO, silk, and silk-CNT substrates before cell seeding (**A**, **B**, **C**, respectively) and after incubating with hESCs for 7 days (**D**, **E**, **F**, respectively). On the silk-CNT surface, there were some micro silk-CNT aggregates distributed within silk matrices (C). After 7 days, the silk-CNT substrate became porous. Some neuronal axons were found to extend into those concaves on the substrate (F). Scale bar, 20 μm.

**Figure 7:**
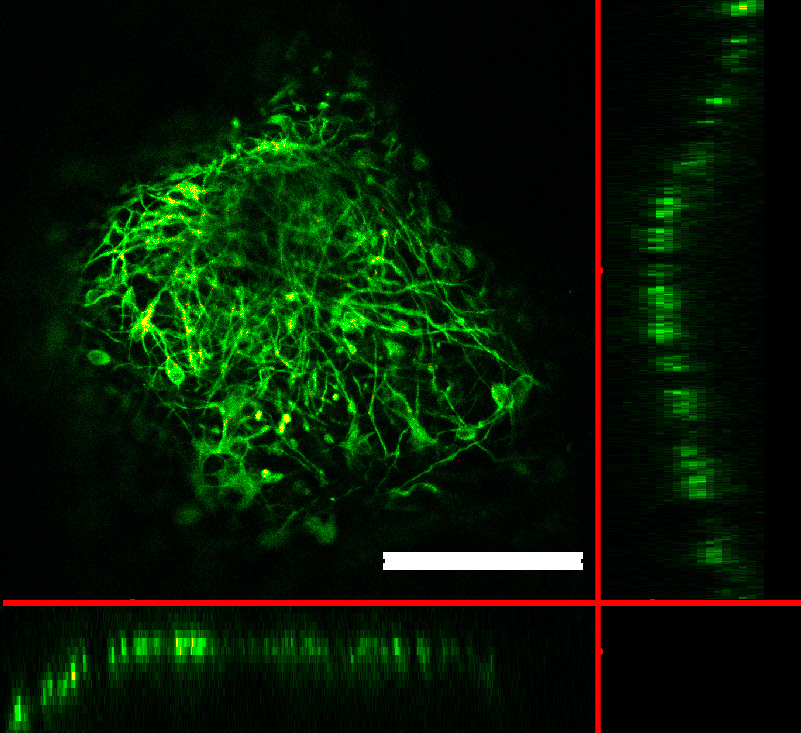
**hESCs on 3D silk-CNT matrix**. Confocal microscopy image of hESC cultured on a silk-CNT scaffold showing β-III tubulin expression in axonal shooting into three-dimensional scaffold matrices. Scale bar, 200 μm.

Both silk and silk-CNT substrates began to degrade and became more brittle during incubation. After 7 days of incubation with hESCs, fibroin fragments were present under light microscopy. We further investigated the substrate degradation with SEM. In contrast to the flat surface properties shown in both PLO and silk substrates, spherical silk-CNT aggregations were found in the silk-CNT composite (Figure 6C). Those silk-CNT microaggregates ranged from a few microns to tens of microns. After 7 days incubation, three-dimensional porous structures were observed on silk-CNT matrices. There were no distinguished microstructures found on either PLO or silk only substrates. Those porous structures of silk-CNT substrates may provide a higher surface area for cell-substrate interactions and sufficient space for neuron axon extension. Results showed that long axons from the soma extended into the space between silk-CNT fragments. In SEM images, we also noticed that some neuronal cells may migrate into the degraded parts of silk-CNT scaffolds (Figure 5 and 6). The degradation of fibroin-based scaffolds could contribute to the infiltration of cells into three-dimensional matrices (Figure 5 and 6). Confocal laser scanning microscopy was used to confirm the three-dimensional cell growths. Differentiated neuronal cells were labeled with β-III tubulin and scanned with optical sectioning (thickness, 0.44 μm) (Figure 7). 3-D reconstructed images indicated that the cells can grow into the silk-CNT matrices for more than 50 μm. This natural biodegradation feature of the silk-CNT substrates promotes the 3-D cell growth, which resembled the more physiological environment than a two-dimensional [2-D] substrate. As a potential vehicle for neural implantations, the biodegradation of silk-CNT substrates also provides additional benefits to minimize unnecessary removal by surgical intervention.

## Conclusions

In this study, our results demonstrated the potential of the silk-CNT composite as scaffolds to support neuronal differentiation for regenerative medicine (Figures 1,2,3,4). The silk-CNT composite scaffold hybridizes advantages from both naturally derived and synthetic materials; fibroin provides a mechanically robust matrix and biodegradable properties for tissue transplantation vehicles [8,13,15,17]. Amphiphilic silk protein here not only provides biodegradable matrices to physically incorporate CNTs in the scaffold, but also acts as an effective dispersant to distribute CNTs homogeneously within the matrix, which is a major limitation for CNT applications within hydrophilic networks. Additionally, CNTs embedded in the silk matrix may promote electron signal transmissions between neurons [10]. In comparison to 2-D PLO substrates, the silk-CNT composite increases neuronal differentiation and provides three-dimensional matrices for cell growth. Further observation showed that hESCs cultured on the silk-CNT scaffold exhibited higher maturity along with dense axonal projections. Our results support silk-CNT scaffolds as one viable candidate for nerve repair treatments of patients suffering from SCI or MS.

## Competing interests

The authors declare that they have no competing interests.

## Authors' contributions

CSC, EYTC, and WCC designed the research. CSC, SS, CL, and MB performed the research. CSC, SS, CL, EF, and MB analyzed the data, and CSC, SS, MB, EYTC, EF, and WCC wrote the paper. All authors read and approved the final manuscript.
